# Supplementary material for: Bereavement practices and staff competencies on perinatal loss at a community-based teaching hospital
Source: PLoS One. 2026 Apr 27;21(4):e0347348. doi: 10.1371/journal.pone.0347348 (PMC13120050; doi:10.1371/journal.pone.0347348)
Supplement: S1 File — (DOCX) [file pone.0347348.s001.docx]

**Supplemental 1: 14 questions that were removed from PBCCS.**

**Bereavement Support Knowledge**

1. I do not know the legal process associated with perinatal loss before 24 weeks gestation.
2. I do not have adequate practical knowledge for bereavement support.
3. I know the legal process associated with perinatal loss after 24 weeks gestation.

**Bereavement Support Skills**

1. I can provide the relevant information required by bereaved parents.
2. I can easily respond to the needs of bereaved parents expecting their next baby.

**Self-Awareness**

1. I am conscious of the particular needs of bereaved parents expecting their next baby.

**Organization Support**

1. I have adequate peer support in my workplace in relation to providing bereavement support.
2. My organization provides bereavement support training.
3. I get recognition for providing effective bereavement support.
4. The workload of the ward/unit hinders effective bereavement support.
5. My workload hinders effective bereavement support.
6. There is adequate number of midwives to cover the ward/unit to enable the provision of bereavement support.
7. My work environment allows me to feel relaxed to carry out my daily work.
8. I find it difficult to ask for support from my workplace management in relation to providing bereavement support.
