# Supplementary material for: Bereavement practices and staff competencies on perinatal loss at a community-based teaching hospital
Source: PLoS One. 2026 Apr 27;21(4):e0347348. doi: 10.1371/journal.pone.0347348 (PMC13120050; doi:10.1371/journal.pone.0347348)
Supplement: Attachment — Submitted filename: Response to Reviewers.docx [file pone.0347348.s003.docx]

PLOS One

Jonathan Bayuo, PhD, Academic Editor, PLOS One

Dear Dr. Bayuo and Dr. Abboah-Offei,

Thank you for the opportunity to revise and re-submit our manuscript PONE-D-25-50589, *Bereavement Practices and Staff Competencies on Perinatal Loss at a Community-Based Teaching Hospital*. We greatly appreciate the feedback received from the editorial office and peer reviewers and made substantial edits and additions to the manuscript accordingly. The tracked and clean versions of this manuscript are uploaded within the revised submission. Please find our point-by-point response to each reviewer’s comment below.

Thank you very much for your time and feedback,

**Suzanne J. Rose, PhD**

Suzanne J. Rose, MS, PhD, CCRC, FACRP (she/hers)

Executive Director of Research, Stamford Health

Assistant Professor in the Department of Medical Sciences, Quinnipiac University

Stamford Health | One Hospital Plaza | Stamford, CT, 06902

Phone: 203.276.7866 | Email: [srose@stamhealth.org](mailto:srose@stamhealth.org)

| Editorial & Reviewer Comments | Author Responses |
| --- | --- |
| Editorial Comments: | |
| When submitting your revision, we need you to address these additional requirements.  1. Please ensure that your manuscript meets PLOS ONE's style requirements, including those for file naming. The PLOS ONE style templates can be found at  https://journals.plos.org/plosone/s/file?id=wjVg/  PLOSOne_formatting_sample_main_body.pdf and  https://journals.plos.org/plosone/s/file?id=ba62/  PLOSOne_formatting_sample_title_authors_affiliations.pdf | Thank you, the paper was edited to reflect PLOS ONE’s style requirements including those for file naming |
| 2. In the online submission form, you indicated that: “All de-identified data is publicly available at the following location: [https://figshare.com/s/1e143cb02cde1e5f4ef4](https://nam04.safelinks.protection.outlook.com/?url=https%3A%2F%2Ffigshare.com%2Fs%2F1e143cb02cde1e5f4ef4&data=05%7C02%7Cjohartnett%40burke.org%7Ca1fa48115b6d48f0bba108de669f4b46%7C9c01f0fd65e040c089a82dfd51e62025%7C0%7C0%7C639061031855088335%7CUnknown%7CTWFpbGZsb3d8eyJFbXB0eU1hcGkiOnRydWUsIlYiOiIwLjAuMDAwMCIsIlAiOiJXaW4zMiIsIkFOIjoiTWFpbCIsIldUIjoyfQ%3D%3D%7C0%7C%7C%7C&sdata=PyCQm9fQLJmrMpfRMJaJRZC4wV9WiRwlrqAhyZrUrYA%3D&reserved=0) and can also be provided with any written request to the corresponding author.”    All PLOS journals now require all data underlying the findings described in their manuscript to be freely available to other researchers, either 1. In a public repository, 2. Within the manuscript itself, or 3. Uploaded as supplementary information.  This policy applies to all data except where public deposition would breach compliance with the protocol approved by your research ethics board. If your data cannot be made publicly available for ethical or legal reasons (e.g., public availability would compromise patient privacy), please explain your reasons on resubmission and your exemption request will be escalated for approval | Thank you for your review and feedback.  All de-identified data is publicly available within *figshare* at the following location: <https://doi.org/10.6084/m9.figshare.30138229> . We confirm that the study dataset is publicly available for download and can be accessed by anyone who clicks the link.  Full citation: Karki, Shweta; Rose, Suzanne (2025). Bereavement Practices and Staff Competencies on Perinatal Loss at a Community-Based Teaching Hospital. Figshare. Dataset. <https://doi.org/10.6084/m9.figshare.30138229.v1> |
| 1. It is unclear why the authors label the study as a "pilot study". With the use of survey, a cross-sectional design will be more appropriate. Kindly consider this further. | Thank you for your comment. We removed the word “pilot” in multiple areas of the manuscript, in addition to the abstract, where we denote this is a cross-sectional survey study. |
| 2. Kindly add the absolute figures to the percentages mentioned in the abstract section. | Thank you for your comment. We added the absolute figures to the percentages mentioned in the abstract. |
| 3. The authors mention 11 questions were removed from the original instrument. It will be helpful, for methodological purposes, to list these questions in a table.  Also, was the validity and reliability scores re-evaluated for the modified scale? This is extremely important. | Thank you for your comment. We present the questions that were either augmented or removed from the current study. The questions can be found in the Supplemental 1 section of the manuscript. Regarding the validity and reliability of the questionnaire, the survey was originally developed to evaluate bereavement confidence, competencies, and skills among midwives, rather than among acute care hospital staff in the United States who also care for patients experiencing perinatal loss. Accordingly, certain items were not applicable to our target population and were reviewed for relevance prior to administration. Our intent was not to re-validate or evaluate the instrument for acute care hospital staff, but rather to use the existing tool pragmatically to assess staff confidence, competencies, and perceived skills within our specific clinical context. This approach is consistent with exploratory or needs-assessment applications in which established instruments are adapted to inform local practice and training priorities. As noted in the manuscript, we obtained permission from the survey’s developer, Dr. Kalu, prior to use, and approval was granted for its implementation in this study; she also reviewed the study findings and provided input on their interpretation. With that said, this was added as a limitation to the current study and can be found in the main text in the clean version from lines 315-321. This change is also in the tracked changes version for reference. |
| 4. The "omission of missing data" could use more details. Does the authors mean all responses that were incomplete were discarded? | Thank you for your comment. Surveys containing missing responses were not excluded from analysis. The questionnaire was intentionally designed so that items were not mandatory, given the sensitive nature of the topic, in order to ensure that participants did not feel compelled to answer every question. We added additional details which can now be found from lines 189-192 in the clean version. This change is also in the tracked changes version for reference. |
| 5. Did the authors observe any variations across the participants from the L&D and ED? Such nuances, if observed, should be highlighted. | Please see Table 1 for demographic breakdown. Table 4 is also included which demonstrates one significant difference between departments in the self-awareness subscale. |
| ***Reviewer #1:*** | |
| This is a well written manuscript with significant findings for improving perinatal loss management in hospital settings as well as highlighting the pressing need for structured training programs and institutional resources to equip staff with the necessary skills to provide compassionate and effective bereavement support. I have only minor comments for clarification purposes.: 1. On page 4 line 109, could you provide the full meaning of the 'TEARDROP' program described as an interdisciplinary training on perinatal bereavement care, if there is. | Thank you for your comment. The TEARDROP program’s Core Elements are below, and details on the abbreviation are included in the main text clean version from lines 107-113. This change is also in the tracked changes version for reference.   - Training staff in bereavement-sensitive communication - Empathy-centered clinical practices - Assessment of family emotional needs - Resources for grief support and counseling - Documentation protocols for loss events - Remembrance activities (memory boxes, photos, keepsakes) - Ongoing follow-up support - Policies to standardize care across departments |
| 2. On page 5 line 128 -129, could you clarify the statement 'This pilot study was conducted after receiving Institutional Review Board review and 'determination as exempt' what do you mean 'determination as exempt'? | This means that the study was reviewed by the Institutional Review Board and received a determination of exemption under 45 CFR 46, as it met criteria for minimal-risk research. |
| 3. On page 6 lines 162-164, 'As the questionnaire was originally developed for midwives and nurses in Ireland, some items (11 total) were not relevant to the current U.S. healthcare system and thus removed for the current research.'  Could you clarify if these 11 items removed were within the same subscale? If so, as part of this tool development, does the developers allow for each subscale to be assessed as a separate component and thus removing these 11 items does impact on the total outcome score for the tool? | We present the questions that were either augmented or removed from the current study. The questions can be found in the [Supplemental 1] section of the manuscript, as well as listed below for your review. Regarding the validity and reliability of the questionnaire, the survey was originally developed to evaluate bereavement confidence, competencies, and skills among midwives, rather than among acute care hospital staff in the United States who also care for patients experiencing perinatal loss. Accordingly, certain items (see Supplemental 1) were not applicable to our target population and were reviewed for relevance prior to administration. Our intent was not to re-validate or evaluate the instrument for acute care hospital staff, but rather to use the existing tool pragmatically to assess staff confidence, competencies, and perceived skills within our specific clinical context. This approach is consistent with exploratory or needs-assessment applications in which established instruments are adapted to inform local practice and training priorities. As noted in the manuscript, we obtained permission from the survey’s developer, Dr. Kalu, prior to use, and approval was granted for its implementation in this study; she also reviewed the study findings and provided input on their interpretation. With that said, this was added as a limitation to the current study and can be found in the main text in the clean version from lines 315-321. This change is also in the tracked changes version for reference. |
